# Neural Correlates of Sexual Orientation in Heterosexual, Bisexual, and Homosexual Men

**DOI:** 10.1038/srep41314

**Published:** 2017-02-01

**Authors:** Adam Safron, David Sylva, Victoria Klimaj, A. M. Rosenthal, Meng Li, Martin Walter, J. Michael Bailey

**Affiliations:** 1 Department of Psychology, Northwestern University, USA; 2 Department of Psychiatry, Kaiser Permanente, USA; 3 Department of Neurology, Otto von Guericke University Magdeburg, Germany

## Abstract

Studies of subjective and genital sexual arousal in monosexual (i.e. heterosexual and homosexual) men have repeatedly found that erotic stimuli depicting men’s preferred sex produce strong responses, whereas erotic stimuli depicting the other sex produce much weaker responses. Inconsistent results have previously been obtained in bisexual men, who have sometimes demonstrated distinctly bisexual responses, but other times demonstrated patterns more similar to those observed in monosexual men. We used fMRI to investigate neural correlates of responses to erotic pictures and videos in heterosexual, bisexual, and homosexual men, ages 25–50. Sixty participants were included in video analyses, and 62 were included in picture analyses. We focused on the ventral striatum (VS), due to its association with incentive motivation. Patterns were consistent with sexual orientation, with heterosexual and homosexual men showing female-favoring and male-favoring responses, respectively. Bisexual men tended to show less differentiation between male and female stimuli. Consistent patterns were observed in the whole brain, including the VS, and also in additional regions such as occipitotemporal, anterior cingulate, and orbitofrontal cortices. This study extends previous findings of gender-specific neural responses in monosexual men, and provides initial evidence for distinct brain activity patterns in bisexual men.

## Sexual Orientation and Sexual Motivation

The term *sexual orientation* is used to indicate an individual’s sexual arousal and attraction patterns, sexual behavior patterns, or sexual identity^1^, which often go together but can also differ from each other^2^,^3^,^4^,^5^,^6^. While most individuals identify as heterosexual, a significant number of individuals also report identifying as homosexual (1.9–2% of the US population) or bisexual (2–4% of the US population), with even greater proportions reporting some degree of same-sex behavior or attraction^7^,^8^.

If the term sexual orientation is used to describe a pattern of arousal and attraction^1^, then genital assessment has high face validity for studying sexual orientation in men. Neuroimaging, however, may have a variety of methodological advantages, including the potential for greater sensitivity in detecting motivational responses to stimuli that are psychologically significant yet unlikely to result in noticeable physiological changes^9^ or even subjective responses^10^. Even with briefly-presented erotic pictures, fMRI has demonstrated a high degree of sensitivity and specificity in measuring sexual orientation^11^,^12^,^13^.

Furthermore, genital arousal is capable only of indicating degree of increase or decrease along a single dimension of tumescence, and thus provides little qualitative information on the mental states underlying sexual arousal and desire. Neuroimaging techniques, in contrast, can localize activity within various brain structures, and therefore suggest and test hypotheses about diverse psychological processes influencing sexuality. The present research focuses on reward-related brain regions.

## Neural Correlates of Sexuality and Reward

Numerous studies have examined the neuroimaging correlates of responses to sexual stimuli since the first investigations by Rauch *et al*.^14^ and Stoléru *et al*.^15^. Specific results vary by experimental paradigm, but findings generally suggest that mechanisms underlying the response to erotic stimuli overlap with those involved in responding to arousing and rewarding stimuli more generally^16^,^17^,^18^. This is unsurprising, since to the extent that individuals are oriented to seek out particular sexual interactions^19^, they are probably—although not necessarily^5^—motivated by the anticipation that such interactions will be rewarding^20^,^21^,^22^.

However, since a particular brain area can activate for multiple reasons, caution is needed in making inferences about functional significance from observed activity^23^,^24^,^25^. When sexual stimuli produce significant fMRI responses, it can be difficult to determine the extent to which different brain regions indicate general arousal, sexual arousal, or both. In studies that have attempted to distinguish sexual arousal and general arousal (i.e., non-sexual and potentially non-valenced autonomic, neuroendocrine, and neuromodulatory changes), only activity within the ventral striatum (VS) and hypothalamus have been specifically associated with the experience of stimuli as erotic, and in addition correlating with degree of sexual intensity^26^,^27^.

The VS—and in particular the nucleus accumbens subsection—is a neural epicenter for selecting actions on the basis of their relative valuations^28^,^29^. As such, it may be the ideal region of interest for investigating sexual preferences. VS activity is the most widely-used measure of preferences for choices and outcomes in the neuroeconomics literature^30^,^31^. The VS has been associated with motivational processes in a wide variety of neuroimaging studies, ranging from the desire to breathe when deprived of air^32^, or drink when thirsty^33^, to cravings for food^34^ and drugs^35^, to feelings of aesthetic appreciation and attraction^36^, to compulsive videogame playing^37^, social approval^38^,^39^, monetary rewards^39^,^40^,^41^, and more. The underlying factor across all of these paradigms is reward, suggesting that the VS contributes to a “common neural currency” of value^42^.

Notably, dopaminergic stimulation of the VS does not produce subjective feelings of pleasure^43^,^44^,^45^, but rather seems to be more closely related to desire, craving, or wanting. Indeed, one study found that relative VS responses to food and erotic images predicted relative levels of weight gain and sexual desire in the months following fMRI assessment^46^.

Thus, while VS activity does not necessarily indicate sexual arousal, it can nonetheless be used to assess relative degrees of motivation while viewing sexual stimuli^28^,^47^,^48^. That is, relatively greater VS activity in response to either male or female erotic images would be consistent with relative androphilic or gynephilic preferences, respectively.

Support for VS as a measure of male sexual orientation is also evidenced by earlier studies of heterosexual and homosexual men’s neural responses to visual erotica^13^,^49^,^50^,^51^,^52^. All of these studies found the VS to be included among brain regions with greater activity in response to erotic stimuli featuring participants’ preferred sexes (i.e. women for heterosexual males and men for homosexual males) relative to their non-preferred sexes (i.e. men for heterosexual males and women for homosexual males).

These neuroimaging results are consistent with findings from the genital arousal literature^53^,^54^,^55^,^56^, wherein heterosexual or homosexual men both tend to exhibit *category-specific* patterns of subjective and genital arousal. That is, heterosexual and homosexual men exhibit—with a high degree of correspondence between genital and subjective measures—substantial arousal to erotic stimuli depicting their preferred sex, and little arousal to their non-preferred sex. Neuroimaging research suggests that these category-specific arousal patterns are also reflected in category-specific patterns of activation in the brain’s reward system^13^,^49^,^50^,^51^,^52^,^57^,^58^.

What do these patterns look like for bisexual men? Studies of bisexually-identified men have produced inconsistent findings^59^,^60^. Bisexual men consistently exhibit bisexual patterns of subjective arousal, but have sometimes shown category-specific patterns of genital arousal^4^,^61^,^62^. More recently, bisexual genital arousal patterns have been found in a study that used particularly strict inclusion criteria for bisexuality, requiring bisexual participants to have had at least two sexual partners and one romantic partner (of three months or greater duration) of each sex^63^,^64^. The present study is the first to study bisexuality using neuroimaging.

## The Present Research

First, we examined the category specificity of sexual motivation among monosexual (i.e., homosexual and heterosexual) men by comparing VS activation patterns to erotic pictures and videos depicting males versus those depicting females. These data were also compared with subjective responses to those same stimuli. Then, we investigated the degree to which bisexual men showed different VS responses to erotic pictures and videos compared with monosexual men. Of note, our bisexual male subjects were a subset of those from our previous genital arousal research (finding support for bisexual arousal patterns) (Rosenthal *et al*.)^63^,^64^. Thus, they comprise a promising sample to investigate neurally. Finally, we explored the degree to which heterosexual, homosexual, and bisexual men exhibited category-specific activation patterns across the entire brain, including neural regions for which we did not have a priori hypotheses. If the VS is a particularly valid indicator of positive incentive value, then we would expect the whole brain analyses to show it to be a relatively robust indicator of sexual orientation.

In all analyses, we examined activation patterns in response to both erotic pictures and videos. Although these patterns can be expected to be largely similar^65^,^66^, picture and video stimuli have different strengths and weaknesses. For example, picture stimuli may be better for exploring the initial appraisal of stimuli, with less influence from higher-order processing^18^,^67^. Video stimuli, in contrast, might be better for exploring deeper emotional states or for assessing the effects of more elaborative processing on sexual responses.

## Method

### Participants

26 heterosexual men, 28 bisexual men, and 25 homosexual men were recruited using internet advertisements on Craigslist. Bisexual participants were required to have had at least two sexual partners and one romantic partner (of three months or greater duration) of each sex, as described in a previous study of genital arousal in this population^63^,^64^. The data described here are from a slightly smaller subset of the men from Rosenthal *et al*.^63^,^64^, which included 28 heterosexual, 30 bisexual, and 27 homosexual men, because two heterosexual, five bisexual, and two homosexual men did not undergo fMRI assessment; three additional bisexual men were identified and participated in the experiment subsequent to our initial publication^63^,^64^.

After responding to advertisements, participants were screened for inclusion using online questionnaires. Participants provided information about sexual orientation, sexual interests, and personality, in addition to answering screening questions relevant to medical eligibility for fMRI research. Participants were informed of the risks and nature of the study, and agreed to participate in questionnaire, fMRI, and genital arousal portions of the research. Genital arousal data were previously reported in Rosenthal *et al*.^63^,^64^. All methods were approved by the Institutional Review Board of Northwestern University and carried out in accordance with its guidelines. Informed consent was obtained from each participant for every portion of the study in which they participated.

Participants’ sexual orientation was assessed using a modified Kinsey score^68^, which asked participants about their sexual fantasies throughout adulthood as well as in the past year^53^,^69^. The scale ranged from 0 to 6, with 0 corresponding to an exclusively heterosexual orientation and 6 corresponding to an exclusively homosexual orientation. Responses to the questions about adulthood and about the past year were averaged to create a Kinsey score for each participant. The average Kinsey score was 0.4 for heterosexual men (*SD* = 0.46, *range* = 0–1.5), 3.2 for bisexual men (*SD* = 0.85, *range* = 2–4.5), and 5.7 for homosexual men (*SD* = 0.45, *range* = 5–6). Self-reported sexual identities (i.e., “Homosexual”/“Gay”, “Bisexual”/“Bi”, “Heterosexual”/“Straight”) corresponded with the Kinsey score ranges for all participants.

Participants’ ages ranged from 25 to 50 years old. Mean ages were 32.3 for heterosexual men (*SD* = 6.75, *range* = 25–48), 37.5 for bisexual men (*SD* = 8.32, *range* = 26–50) and 33.2 for homosexual men (*SD* = 6.4, *rang*e = 26–50). The sample was racially and ethnically diverse, with Caucasian (60.76%), Latino (10.13%), African American (12.66%), and Asian American (6.33%) participants, as well as participants who identified otherwise or who identified as multiracial (10.13%).

## Stimuli and Procedure

### Stimuli and assessment

#### Picture runs

The present study employed a subset of the picture stimuli used in Safron *et al*.^13^ and Sylva *et al*.^70^. Pictures depicted a nude man, a nude woman, or a same-sex couple (i.e, either two men or two women) engaged in explicit sexual contact. Erotic stimuli featuring same-sex pairs engaging in explicit sexual interaction is common in research on sexual arousal and sexual orientation^53^,^61^,^69^. Such stimuli are similar to pictures of nude individuals, in the sense that only men or women, but not both, are depicted in a given picture. Thus, sexual arousal induced by them is relatively unambiguous. However, erotic stimuli featuring explicit sexual activity in couples tends to be substantially more arousing compared with pictures of nudes^69^.

In each of two 10.5-minute runs (ordering counterbalanced), participants viewed 40 erotic pictures featuring male actors and 40 erotic pictures featuring female actors. Each picture was shown for 3.5 seconds, followed by a variable-duration fixation cross presented for either 1.5, 6.5, or 11.5 seconds. During the presentation of each picture, participants used buttons held in their right hands to rate that image on a scale of −2 to +2 (respectively: “strongly disliked,” “disliked,” “liked,” “strongly liked”), with no option of 0 for neutral ratings.

#### Video runs

Following picture assessment, participants were shown six video clips depicting individual masturbating men and six video clips depicting individual masturbating women. Depicted individuals appeared sexually aroused but did not reach orgasm. To estimate baseline responses, six natural landscape videos were shown.

In each of two 9.25-minute runs (ordering counterbalanced), videos were presented for 15 seconds each, followed by a 15-second distraction task requiring participants to indicate via button-press when a number in a series decreased by an interval other than seven. This task was intended to facilitate a return to emotional and physiological baseline.

After leaving the scanner, participants viewed the videos once more and provided ratings of each clip. Videos were rated using a 5-point scale for degree of sexual appeal, ranging from “not at all” (0) to “very much” (4), with a midpoint of “somewhat” (2).

### fMRI signal extraction methods

#### Image acquisition

A Siemens Trio 3 T magnet and 12-channel RF head coil were used to collect T2*-weighted gradient-recalled EPI images from the whole brain (32 3-mm slices with a 0.99-mm interslice gap; TR = 2500 ms; TE = 20 ms; flip angle = 80°; FOV = 200 × 220 mm, 120 × 128 matrix). Slices were taken along the plane connecting the anterior and posterior commissures, with a 1.72 mm × 1.72 mm × 3.99 mm resolution, with more refined axial dimensions intended to produce less distortion and signal dropout in sub-cortical areas, although possibly at the expense of signal-to-noise ratio. During each picture run, 250 whole-brain volumes were collected, and during each video run, 220 whole-brain volumes were collected, with the first four volumes discarded to account for initial magnetization effects. For anatomical localization, a structural MRI scan consisting of T1-weighted images was conducted after the testing runs (160 1-mm axial slices; TR = 2.1 ms; TE = 4.38 ms; flip angle = 15°; FOV = 220 mm; 256 × 192 matrix).

#### Image pre-processing

Image pre-processing and analysis was performed using SPM 12b (Wellcome Trust Centre for Neuroimaging, London, UK)^71^, and implemented in Matlab v 8.1.604 (The MathWorks Inc., MA, USA).

Functional (EPI) volumes were first corrected for slice timing. Each participant’s volumes were then registered to the mean slice, after which the registered volumes were resliced, used to create a mean resliced image, and then co-registered to the mean structural (T1) image. All EPI images, including the mean resliced image, as well as the structural (T1) scans were then spatially normalized to Montreal Neurological Institute (MNI) space, and re-sampled to 3 × 3 × 3 mm (27 mm^3^) resolution. Normalized functional images were then smoothed to an 8 mm full-width-at-half-maximum Gaussian kernel.

#### Signal to noise ratio and head coverage exclusions

To exclude participants with poor signal due to either head motion or scanner conditions, average signal-to-noise ratio (SNR) over time was calculated for each subject (after preprocessing, using a mask that included only voxels with appreciable EPI signal). The SNR ratio for each voxel (mean divided by standard deviation) was averaged across all voxels in the brain^72^,^73^. Participants whose picture data SNR was less than one standard deviation below the mean were excluded from picture analyses. Similarly, participants whose video data SNR was less than one standard deviation below the mean were excluded from video analyses.

Based on these criteria, eleven participants (two heterosexual, seven bisexual, and two homosexual) were excluded from fMRI and subjective picture analyses, and ten participants (four heterosexual, five bisexual, and one homosexual) were excluded from fMRI and subjective video analyses. An additional six participants (one heterosexual, four bisexual, and one homosexual) were excluded from subjective picture rating analyses due to insufficient subjective data resulting from a data-recording error. An additional five participants (three heterosexual, four bisexual, and two homosexual) were excluded from subjective video rating analyses for the same reason. Thus, after exclusions were performed for both SNR and insufficient subjective data, we included a total of 23 heterosexual men, 17 bisexual men, and 22 homosexual men in picture analyses, and included 19 heterosexual men, 19 bisexual men, and 22 homosexual men in video analyses.

To check the validity of our SNR-exclusion criterion, head motion plots were visually inspected for all participants^72^. Excluded participants were confirmed to have highly variable head positions as compared to included participants. An additional validity-check was performed using evoked responses to erotic pictures minus a fixation-cross baseline. Excluded participants had substantially reduced activity in visual cortices as compared to included participants.

For whole-brain analyses, mean functional scans were individually examined to identify participants with substantial cutoffs in head coverage. As a result, one homosexual male who had substantial frontal lobe cutoff was excluded from whole-brain analyses in addition to those participants excluded for SNR.

#### First-level analyses

For both the video and picture assessments, a standard general linear model (GLM)^71^ was used to identify hemodynamic changes for each participant, and a high-pass filter (cutoff 128 s) was used to remove low-frequency temporal noise.

For the picture assessment, each participant’s responses to each stimulus contrast of interest were concatenated within stimulus type, using data from both runs. Estimated average activity was calculated for each participant’s separate responses to male pictures, female pictures, male videos, and female videos (contrasted with fixation cross for pictures and nature scenes for videos). These estimates were used for region of interest analyses. For whole-brain analyses, estimated average activity was also calculated for each participant’s response to male compared with female pictures and videos.

#### Ventral striatum region of interest analyses

An a priori region of interest (ROI) analysis was performed on the VS—centered on the nucleus accumbens—as this was the area most likely to indicate reward. The VS and hypothalamus are the only two areas that have been shown to be specifically associated with sexual (as opposed to general) arousal^26^,^27^. We focused on the VS because it likely has higher validity for reflecting reward compared with the hypothalamus, which contains a variety of nuclei with heterogeneous functions (including sexual arousal) that would be difficult to disambiguate with the limited spatial resolution of 3 T fMRI.

**Figure 1:**
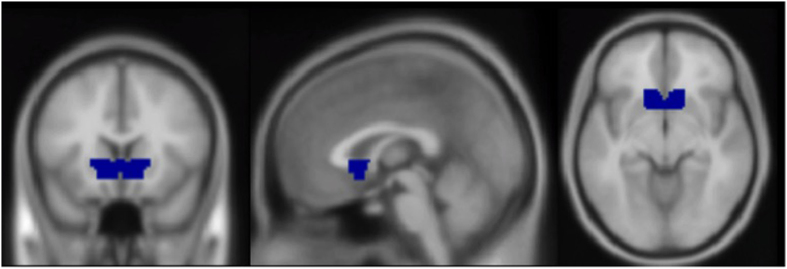
Mask used as the ventral striatum (VS) ROI, drawn using an average brain in the WFU PickAtlas toolbox for SPM 8. MNI coordinates displayed: x = 0, y = 17, z = −8.

The VS ROI mask used in the present study was drawn on an MNI template brain using the WFU PickAtlas toolbox for SPM 8^74^. It was anatomically defined as a dilated intersection of the ventral anterior caudate and putamen. The resulting VS ROI is shown in Fig. 1.

Estimates of average VS activity for each participant were extracted using the MarsBar toolbox for SPM8^75^. Extracted VS ROI data were analyzed using JMP Pro v11 (SAS Institute, Cary, NC).

### Planned contrasts

#### Category-specificity in heterosexual and homosexual men

First we examined how well VS activity predicts sexual orientation in monosexual men via differential responding to male versus female erotic stimuli within our paradigm. For each participant we separately calculated differential VS activation for erotic pictures and videos by subtracting activation to female erotic stimuli from activation to male erotic stimuli. The straightforward prediction is that on average, these contrasts should yield positive values for homosexual men and negative values for heterosexual men.

#### Comparing bisexual and monosexual men

What comprises evidence for bisexual neural activation patterns in men? In exploring whether bisexually-identified men have greater propensity to bisexual responding compared with monosexual men, it is inadequate to show that on average, bisexual men tend to exhibit significant VS activity to both sexes. This is because a group average showing a high response to both sexes could be the result of particular members of the group responding strongly to women and others to men, even if no individual in the group is attracted to both. This would happen if a sample of bisexually-identified men comprised a mixture of men who individually have either heterosexual or homosexual response patterns.

An adequate test of the hypothesis that bisexual-men are uniquely bisexual in their response patterns requires a dependent variable that gauges bisexual responses within individuals. Two related dependent variables have been proposed. The more intuitive is that compared with monosexual men, bisexual men should show a smaller difference, on average, between their responses to men and women^63^,^64^. The dependent variable in this case is the *absolute value of the difference between responses to men and responses to women*; henceforth we refer to this variable as *|Male–Female|*. A slightly different test is motivated by recognizing that regardless of which sex a bisexual man responds less to, he should respond more to that sex compared with a monosexual man’s response to his less activating sex^61^. This test uses *response to the less activating sex as its dependent variable*; henceforth we refer to this variable as *Minimum(Male, Female)*. The two tests provide complementary information, and ideally both should be analyzed in studies of bisexually-identified men’s arousal patterns. These analyses were conducted via one-way ANOVA, implemented via a single planned contrast within a multiple regression model (Judd, McClelland, & Ryan, 2011). Each planned contrast compared the mean of the bisexual subsample with the unweighted pooled means of the other two subsamples.

### Whole brain analyses

Finally, we examined overall patterns of differential activation in response to male compared with female erotic stimuli across the entire brain. If the VS is a particularly valid indicator of desire, then it is also likely to be a particularly robust discriminator of male and female sexual stimuli in monosexual men in whole brain contrasts. Further, if bisexual men have less specific arousal patterns, then they are likely to exhibit less extensive differential activity between male and female stimuli compared with the activity patterns expected for heterosexual and homosexual men. However, with respect to establishing bisexual patterns of brain activity, it is important to remember methodological limitations discussed in the previous section. Accordingly, instead of being used as strong tests of bisexual arousal, these whole brain tests could provide information on average responses to male or female stimuli, with an additional possibility that bisexual men may recruit a unique set of neural regions in differentiating between male and female stimuli.

Tests of average group responses to stimulus conditions were performed using one-sample contrasts. Each group (heterosexual men, bisexual men, and homosexual men) was tested individually for clusters of greater activity for male stimuli compared with female stimuli, and female stimuli compared with male stimuli for pictures and videos separately, using a corrected statistical threshold (*p* < 0.05 FWE).

For these analyses, cluster reports were generated in SPM. Peak activations and cluster extents (extent threshold k = 5) were visually examined as overlays on slice and render maps. Neuroanatomical descriptions were determined based on agreement between two trained investigators, and checked against designations from the WFU Atlas^74^.

## Results

### Planned Contrasts

#### Category-specificity of heterosexual and homosexual men

Consistent with our prediction, there were large and statistically significant VS activation differences between homosexual and heterosexual men when male–female stimuli difference scores were compared (Table 1). For both standardized subjective ratings and VS responses, differences between male and female stimuli (both picture and video) were in the expected directions, with negative scores for heterosexual men and positive scores for homosexual men. These results, consistent with our prediction for all stimulus types, support the VS’s ability to signify sexual orientation for the monosexual men within our sample. If these results had not been consistent with our predictions, they may have called to question the appropriateness of either our ROI or our stimuli for our subsequent tests of bisexual men.

#### Comparing bisexual and monosexual men

Consistent with our predictions (Table 2), bisexual men rated their subjectively less preferred sex significantly higher compared with the ratings of monosexual men (measured using the test Minimum(Male, Female)); further, bisexual men rated male and female stimuli significantly more similarly than did monosexual men (measured using the test |Male–Female|). These results were present for both picture and video stimuli.

VS activation results were somewhat more complicated. Compared with monosexual men, bisexual men showed significantly higher VS responses to their less activating sex for video stimuli, but not for pictures. Bisexual men also showed significantly more similar VS responses between male and female stimuli for pictures but not for videos.

**Figure 2:**
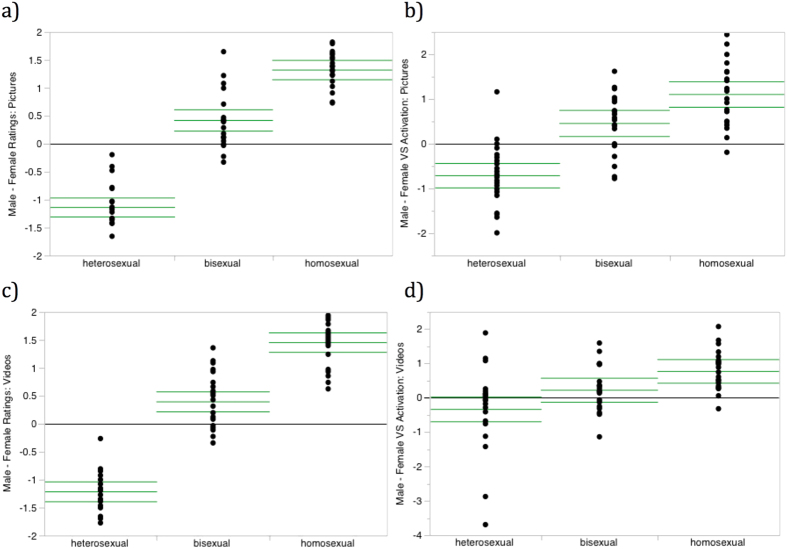
Male-female stimuli difference scores for subjective ratings and VS responses by sexual orientation. Difference scores are defined as a participant’s average response to stimuli depicting males minus average response to stimuli depicting females. Points represent individual participants. Horizontal bars indicate group means and 95% confidence interval of the mean. Horizontal lines at 0 indicate no difference between ratings to erotic stimuli depicting each sex. (**a**) Difference scores for subjective ratings of picture stimuli. (**b**) Difference scores for VS activation evoked by picture stimuli. (**c**) Difference scores for subjective ratings of videos stimuli. (**d**) Difference scores for VS activation evoked by video stimuli.

Individual-level male-female stimuli difference scores are shown for all participant groups in Fig. 2. Patterns are consistent with the contrasts described above, with predominantly positive (male favoring) difference scores in homosexual men, and predominantly negative (female favoring) difference scores in heterosexual men. Overall stimulus differentiation was smaller among bisexual men, but with a slightly greater subjective preference and VS response toward male stimuli, consistent with the Kinsey scores reported by our particular bisexual sample (*M* = 3.2, *SD* = 0.85, *range* = 2–4.5).

To test whether indicators of bisexual activation were significant when considered in aggregate, we regressed the dichotomous variable “Bisexual versus Monosexual” on the four indicators: activation to the less activating stimuli (higher in men with a relatively bisexual pattern) and absolute difference of male and female stimuli (lower in men with a relatively bisexual pattern), measured separately for pictures and videos. Together, the four predictors did not reach statistical significance, whether the dependent variable was treated numerically (via least squares regression) or nominally (via logistic regression): respectively, *F*(4, 59) = 1.82, *p* = 0.14, *R*^2^ = 0.11; χ^2^(4) = 7.55, *p* = 0.11.

### Whole Brain Analyses

*Note: as previously discussed, only the ROI analyses constitute an unambiguous test of bisexual response*.

**Figure 3:**
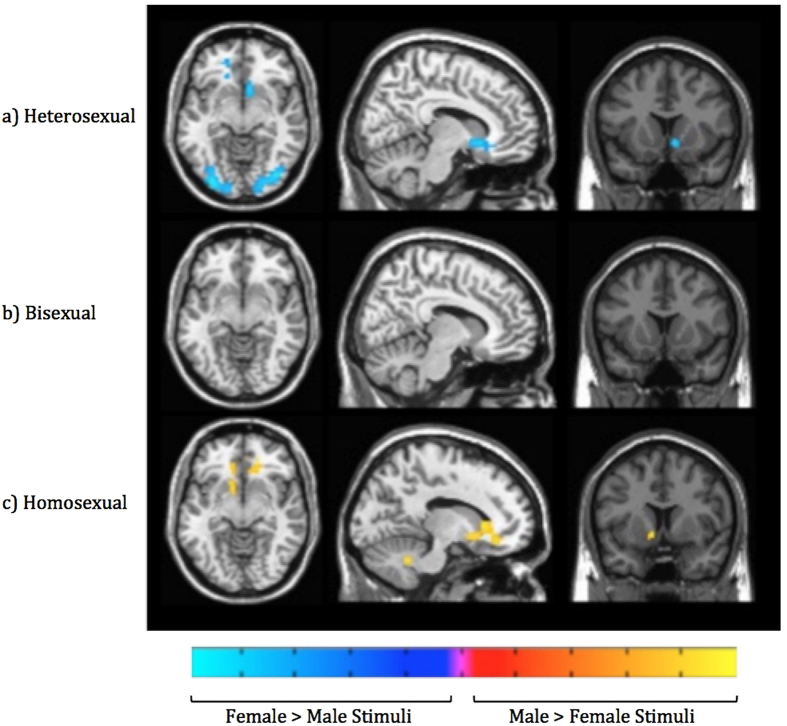
Whole brain activations for the male minus female contrasts when brain activation evoked by viewing neutral stimuli was subtracted from activation toward erotic pictures. Height threshold is set at p < 0.05 FWE with a cluster threshold of k = 5. Axial slice 32 is shown for heterosexual, bisexual, and homosexual men. Sagittal slice 50 is shown for heterosexual and bisexual men, and sagittal slice 39 is shown for homosexual men to facilitate comparisons with heterosexual men’s patterns and to show ventral striatum activation present on the left rather than right side. Coronal slice 38 is shown for heterosexual and bisexual men, and coronal slice 41 is shown for homosexual men in order to display ventral striatum activity and facilitate comparisons with heterosexual men’s patterns.

When responses to male and female erotic pictures were compared (Fig. 3 and Table 3), bisexual men did not exhibit significant differences in their responses to male vs. female stimuli. In contrast, heterosexual and homosexual men showed strong (and opposite direction) differential activations between male and female pictures in multiple cortical and subcortical areas, including the VS.

**Figure 4:**
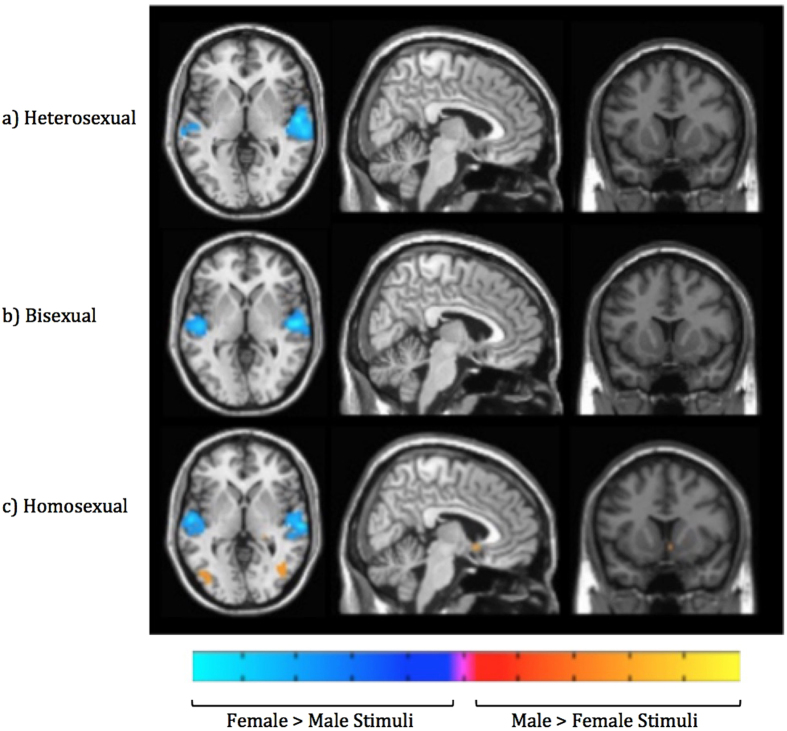
Whole brain activations for the male minus female contrasts when brain activation evoked by viewing neutral stimuli was subtracted from activation toward erotic videos. Height threshold is set at p < 0.05 FWE with a cluster threshold of k = 5. Axial slice 36, sagittal slice 48, and coronal slice 39 are shown for all groups.

When responses to male and female erotic videos were compared (Fig. 4 and Table 4), all groups showed bilateral superior temporal cortex activations consistent with a stimulus confound in which more extensive and substantial vocalizations were present in female (compared with male) erotic videos^76^. Complex parietal activations were observed in bisexual and homosexual groups where some areas showed greater activity for male videos, and others showed greater activity for female videos. Only homosexual men exhibited significantly different VS activation in response to their sexually preferred video stimuli.

## Discussion

In the present investigation, patterns of brain activation were generally consistent with those of previous research in showing that heterosexual and homosexual men’s responses to male and female erotic stimuli are quite distinct^4^,^53^,^54^. Furthermore, our results are consistent with recent past studies of male bisexuality (e.g. Rosenthal *et al*.^63^,^64^) in showing that bisexual men’s responses to male and female stimuli tend to be less distinct compared with monosexual men’s responses^63^,^64^. Here, however, these response patterns were demonstrated using fMRI activations in the ventral striatum (VS), providing an objective measure of valence.

### Category Specificity in Heterosexual and Homosexual Men

Heterosexual men showed greater VS responses to erotic stimuli featuring women, and homosexual men showed greater VS responses to erotic stimuli featuring men. Further, these responses were consistent with subjective assessments of the same stimuli.

Compared with videos, pictures produced somewhat stronger differential VS responses between male and female stimuli, as indicated by larger effect sizes in monosexual region of interest analyses (Table 1) and the robustness of whole-brain findings. One potential explanation for this difference may be that the reliability of the pictures paradigm benefited from the larger number of stimuli used. Alternatively, it may be the case that the VS is more sensitive to briefly presented stimuli, which is consistent with both reward-prediction error^77^,^78^,^79^,^80^ as well as incentive salience models^43^ of dopamine and VS functioning. Indeed, while the VS is frequently active during both reward anticipation and consumption, the association is particularly strong when consumed rewards occur unexpectedly or are uncertain^30^, as might be the case for randomly presented pictures. Additionally, for some participants it could have been the case that wanting or desire responses were strongest at the beginning of a video presentation, but then decreased—or fluctuated—over the subsequent 15 seconds.

### Comparing Bisexual and Monosexual Men

Compared with monosexual men, bisexual men reported relatively bisexual subjective response patterns for all stimuli. With respect to VS activity, significantly bisexual patterns were observed for both picture and video stimuli, but in different tests. Compared with monosexual men, bisexual men showed significantly greater VS activity to their less activating sex for video stimuli, but not for pictures. Bisexual men also showed significantly less differential activation between male and female sexual stimuli for picture stimuli, but not for videos. Thus, although not all relevant fMRI test results were statistically significant, overall VS activation patterns support the idea that the bisexual men had a more bisexual reward responses compared with the monosexual men.

### Whole brain activation patterns

This study focused on the VS as the primary region of interest, but whole brain activation patterns in each group revealed similar category-specific responses in monosexual men, as well as non-specific responses in bisexual men. These findings are described below along with tentative reverse inferences regarding potential functional significances.

### Neural responses to erotic pictures

Heterosexual men had no areas where male pictures produced significantly greater activity than did female pictures. The reverse direction subtraction of female > male pictures, however, revealed extensive activations spanning multiple brain areas. Activity in the right VS likely indicated positive incentive value towards female stimuli (see introduction). This focal VS cluster in the nucleus accumbens exhibited the most robust differential activation compared with all other clusters in the brain, outside of occipital cortex, which likely indicated visual attention^81^,^82^. Right fusiform and adjacent extrastriate cortices may have indicated face and body perception^83^,^84^,^85^. Subgenual anterior cingulate and left orbitofrontal cortices may have indicated positive valence^27^,^86^,^87^,^88^. Additionally, motor strip (i.e. precentral gyrus) may have indicated preparation of motor sequences or perhaps motor imagery^89^,^90^,^91^,^92^.

Similarly to the heterosexual group, homosexual men had no areas where nonpreferred pictures (i.e., female erotic images) produced significantly greater activity than did male pictures. The reverse direction subtraction of male > female pictures, however revealed extensive activations spanning multiple brain areas. Similarly to the results for heterosexual men, some of the most robust activity was observed in the VS (again, focally in the nucleus accumbens), albeit more bilaterally, and particularly in the left hemisphere. Anterior cingulate and medial orbitofrontal cortices may have indicated positive valence^27^,^86^,^87^,^88^. Bilateral caudate body and cerebellum may have indicated imagination and preparation of motor sequences^90^,^93^,^94^,^95^,^96^.

In contrast to the activation patterns obtained for heterosexual men, the bisexual group had no brain areas with significantly different activation between male and female erotic pictures. While this sort of pattern could result from averaging over a mixture of heterosexual and homosexual response profiles, evidence for distinctly bisexuality responses is suggested by region of interest analyses in the VS.

### Neural responses to erotic videos

Heterosexual men had no areas where male videos produced significantly greater activity than did female videos. The reverse direction subtraction of female > male videos, however, revealed activations in bilateral superior temporal cortices, which likely indicated an auditory confound from more extensive and substantial vocalizations being present in female erotic videos^76^.

Similarly to heterosexual men, homosexual men showed auditory activations in response to female compared with male stimuli. However, they also showed activity in the parietal lobe toward female compared with male stimuli, with clusters observed in both the right angular and right supramarginal gyri. The functional significance of these activations is unclear, but could be tentatively interpreted as indicating either mentalizing or mirroring-related-cognition^97^,^98^,^99^,^100^,^101^,^102^,^103^.

More extensive activations were observed in homosexual men for areas of the brain responding more to male than female erotic videos. A small focal cluster was observed in the medial orbitofrontal cortex, which may indicate positive valence^86^. Activity was observed bilaterally (albeit was somewhat right-lateralized) in a larger cluster spanning both early and higher order visual areas including fusiform and extrastriate cortices, and parietal areas indicative of spatial attention, mental imagery, bodily sensations, and possibly mirroring^84^,^85^,^97^,^98^,^99^,^100^,^101^,^102^,^103^. Bilateral (but primarily right-lateralized) pre-motor cortex potentially indicated imagination and preparation of motor sequences^90^. It is unclear why homosexual men showed more extensive category-specific responses than did heterosexual men in response to erotic videos.

In contrast to the patterns obtained for heterosexual and homosexual men viewing erotic videos, bisexual men showed extensive activation patterns for both the female > male and male > female subtractions. For the female > male subtraction, similar auditory-related activity was observed as in heterosexual and homosexual men. An additional parietal cluster was observed in the right angular gyrus, with possible interpretations including mentalizing^97^,^98^,^99^,^100^,^101^,^102^,^103^. For the male > female subtraction, activity in the superior occipital cortex may have indicated visual attention^81^,^82^. Activity in the postcentral sulcus may have indicated bodily sensations^105^,^106^. Additionally, superior parietal lobule and supramarginal gyrus—more anteriodorsomedial than observed in the female > male contrast for homosexual men—may have indicated perception or imagination related to depicted sexual activities^107^,^108^. For bisexual men, erotic videos did not produce clear category-specific brain activity in one direction or another. It is further notable that there were significant findings not present for either heterosexual or homosexual participants, suggesting that bisexuals represent a group with distinct response profiles.

### Limitations

While our initial sample size was large by the standards of neuroimaging studies of human sexuality, a number of subjects were excluded in order to ensure data quality. Consequently, our study may have been somewhat underpowered for detecting small (but potentially meaningful) effects.

The establishment of an appropriate baseline for activation represents an ongoing challenge for studies of the neural bases of sexual responses. An ideal baseline should produce similar affective (and cognitive) responses between groups. While fixation cross presentations and nature scenes likely represented adequate control conditions for erotic pictures and videos (respectively), some participants could have had significant processing differences during these baseline measurements. For example, since we did not measure genital responses in the scanner, we cannot rule out the possibility that some participants (and perhaps in particular bisexual participants) experienced significant erectile activity that continued after the presentation of arousing stimuli. This could have had the effect of reducing the magnitude of evoked responses for our minimum arousal tests.

Future work may benefit from using other kinds of appetitive stimuli as controls (e.g. appealing food or monetary rewards). This could potentially provide an overall measure of reward sensitivity. Additionally, these appetitive stimuli could also be used to create individually customized functional regions of interest. Theoretically, by using a localizer task to determine the most strongly reward-sensitive voxels in each person’s brain, it may be possible to generate a particularly sensitive measure of erotic preferences.

### Future Directions

Consistency among neural measures of incentive value, subjective and genital arousal, and sexual identity supports the idea that male sexual orientation (in heterosexual, bisexual, and homosexual men) is motivated by patterns of sexual arousal and attraction^1^. One question that remains to be examined is the stability of these neural responses over time, both in terms of early development^109^, as well as throughout life^110^. Although it may seem ethically problematic to conduct such research in younger participants, one advantage of fMRI over other measures of sexual interest is the ability to both objectively and non-invasively measure valenced responses to non-explicit stimuli^109^,^111^.

It may be the case that attraction patterns (and patterns of VS activation to male versus female stimuli) of bisexual individuals change based on a variety of contextual factors^112^. Conceivably, for example, a bisexual person may exhibit more gynephilic or androphilic preferences depending on whether they have recently been romantically or sexually involved with women or men. Research measuring individuals over multiple time-points is needed to examine this (admittedly speculative) possibility. Furthermore, it would be especially informative to include fMRI measures alongside measures of genital and subjective responses in a longitudinal study.

In the present study, we focused on the VS as a measure of positive incentive value. Exploring the role of sexual aversion may also be an informative avenue for future investigations^113^. For example, is bisexuality partially related to lower levels of sexual aversion? A major challenge for conducting these investigations would be finding a brain area in which activity is specific to aversion, rather than general arousal or overall salience. In the past, regions such as the anterior insula activity have been used as a metric of disgust^114^, when general visceral emotionality would likely be a more valid inference with respect to this region^115^. Additionally—and not limited in its applications to studying aversion—multi-voxel classifier techniques might be valuable for inferring valence from more sparsely distributed activation patterns^116^,^117^,^118^.

Future investigations should also explore the degree of specificity of reward-related (and possibly aversion-related) brain activity in women of different sexual orientations. Women’s subjective and genital responses to erotic stimuli suggest that relatively bisexual patterns may be observed in women compared to men^53^,^69^,^119^. Bisexual erotic responses in women are further suggested by preliminary fMRI evidence in which monosexual women—contrasted with monosexual men—exhibited greater activity toward non-preferred erotic pictures in posterolateral putamen, dorsal striatum, and thalamus^70^. Functional neuroimaging focusing on the VS may be a particularly valuable converging line of evidence for studying sexual responses in women, as physiological arousal patterns have been difficult to interpret in light of their low correlation with subjective report^120^. In addition, neuroimaging may have unique advantages for directly comparing men and women in their sexual response patterns, as the same type of objective measurement can be performed on the same brain structures in both men and women.

### Conclusion

Here we replicated previous findings of category-specific neural responses in heterosexual and homosexual men, and further provided evidence that bisexual men exhibit distinctly bisexual neural responses. This sample exhibited bisexual arousal patterns in prior analyses of subjective and genital measures^63^,^64^, and has now been shown to exhibit relatively bisexual ventral striatum (VS) activation patterns. Although mixed results have been obtained in studies of genital arousal patterns in bisexual men, this investigation found bisexual patterns of VS activity in a sample in which participants had been specifically screened for bisexual romantic and sexual histories. However, it should be noted that these findings were variable across different tests, with some tests failing to find evidence for distinct patterns in bisexual participants. Nonetheless, convergence between multiple modalities suggests that for the men studied here, bisexual identities are consistent with bisexual patterns of arousal and desire, similarly to monosexual men.

## Additional Information

**How to cite this article:** Safron, A. *et al*. Neural Correlates of Sexual Orientation in Heterosexual, Bisexual, and Homosexual Men. *Sci. Rep.* **7**, 41314; doi: 10.1038/srep41314 (2017).

**Publisher's note:** Springer Nature remains neutral with regard to jurisdictional claims in published maps and institutional affiliations.

## Figures and Tables

**Table 1:** Male-Female Stimulus Contrasts in Heterosexual and Homosexual Men.

	Heterosexual Men	Homosexual Men	*t (df)*	Cohen’s d
Subjective Ratings: Pictures	Mean: −1.141 SD: 0.381	Mean: 1.317 SD: 0.304	23.863** (43)	−9.095
Subjective Ratings: Videos	Mean: −1.219 SD: 0.359	Mean: 1.450 SD: 0.385	24.010** (43)	−7.170
VS Activity: Pictures	Mean: −0.715 SD: 0.665	Mean: 1.100 SD: 0.692	9.067** (44)	−2.674
VS Activity: Videos	Mean: −0.341 SD: 1.210	Mean: 0.767 SD: 0.561	4.040** (44)	−1.175

Male-female stimulus difference scores by sexual orientation of monosexual participants for both standardized subjective ratings and standardized VS activations. Positive difference scores indicate subjective ratings or VS activation greater toward male stimuli. df = degrees of freedom. SD = Standard deviation. **Significant at p < 0.001.

**Table 2:** Planned contrasts comparing bisexual and monosexual men’s subjective responses and VS activation toward both pictures and videos.

	Contrast 1: Minimum (Male, Female)		Contrast 2: |Male – Female|	
	*β*	*t (df)*	*β*	*t (df)*
Subjective Ratings: Pictures	0.979	5.869** (60)	1.53	7.584** (60)
Subjective Ratings: Videos	1.30	8.495** (64)	1.526	9.153** (64)
VS Activity: Pictures	0.029	0.149 (64)	0.463	2.064* (64)
VS Activity: Videos	0.365	2.108* (66)	0.361	1.566 (66)

For Minimum(Male,Female) contrasts, positive betas indicate greater response values in bisexual (vs. non bisexual) men. For the |Male – female| test, positive betas indicate greater response values in monosexual (vs. bisexual) men. df = degrees of freedom. *Significant at p < 0.05. **Significant at p < 0.001.

**Table 3:** Differential brain activations between male and female pictures in heterosexual, bisexual and homosexual men.

R/L	Region	BA	x/y/z	voxels	peak T
Heterosexual Men					
Female > Male Pictures					
R	middle occipital gyrus	18	(33, −85, −1)	313	10.07
R	middle occipital gyrus	18	(27, −91, 11)		8.56
R	primary visual cortex, cuneus, fusiform gyrus, posterior lingual gyrus	17	(21, −94, −1)		8.53
L	middle occipital gyrus	18	(−30, −76, −1)	323	9.5
L	middle occipital gyrus	18	(−30, −88, −7)		9.43
L	primary visual cortex, cuneus	17	(−12, −97, −1)		7.85
R	ventral striatum		(9, 17, −7)	53	8.14
R	subgenual anterior cingulate	32	(15, 35, −10)		7.2
R	subgenual anterior cingulate	32, 25	(9, 26, −10)		6.53
L	orbitofrontal cortex	10	(−18, 44, −7)	19	7.16
L	precentral gyrus	4	(−42, −25, 65)	24	6.94
Male > Female Pictures: no activations					
Bisexual Men					
Female > Male Pictures: no activations					
Male > Female Pictures: no activations					
Homosexual Men					
Female > Male Pictures: no activations					
Male > Female Pictures					
L	left medial prefrontal cortex	10	(−18, 32, 5)	218	9.19
L	ventral striatum, anterior cingulate, medial orbitofrontal cortex	32, 11	(−9, 23, 2)		9.01
R	anterior cingulate, medial orbitofrontal cortex, ventral striatum	32, 11	(21, 32, 8)		8.08
R	caudate body		(18, −10, 26)	47	8.79
L	caudate body		(−21, −10, 26)	36	8.76
L	dentate nucleus		(−12, −52, −31)	23	8.65
R	nodule of vermis		(3, −52, −34)		6.73
L	cerebellar tonsil		(−9, −52, −40)		6.49
R/L	culmen, declive		(0, −58, −19)	44	7.96
R	medial orbitofrontal cortex	11	(6, 44, −13)	15	7.94

Results are presented separately for each orientation group. L = left, R = right, BA = Brodmann area. Coordinates are in MNI space. All clusters were significant with p < 0.05 FWE corrections.

**Table 4:** Differential brain activations between male and female videos in heterosexual, bisexual and homosexual men.

R/L	Region	BA	x/y/z	voxels	peak T
Heterosexual Men					
Female > Male Videos					
R	superior temporal gyrus, secondary auditory cortex, primary auditory cortex	41, 42, 22	(60, −10, 2)	301	11.64
R	superior temporal gyrus, secondary auditory cortex	22	(63, −22, 2)		10.63
R	superior temporal gyrus, primary auditory cortex	41, 42	(51, −22, −1)		8
L	superior temporal gyrus, primary auditory cortex, secondary auditory cortex	41, 42	(−51, −22, 5)	124	11.36
L	superior temporal gyrus, primary auditory cortex, secondary auditory cortex	41, 42	(−66, −25, 2)		8.47
L	superior temporal gyrus, secondary auditory cortex	41, 42	(−63, −31, 11)		6.4
Male > Female Videos: no activations					
Bisexual Men					
Female > Male Videos					
R	superior temporal gyrus, primary auditory cortex, secondary auditory cortex	41, 42, 22	(57, −13, −1)	298	13.08
R	superior temporal gyrus, primary auditory cortex, secondary auditory cortex	42 22	(51, −25, 8)		10.57
R	superior temporal gyrus, secondary auditory cortex	22	(63, −7, −4)		10.09
L	superior temporal gyrus, primary auditory cortex, secondary auditory cortex	41, 42	(−45, −22, 8)	292	10.55
L	primrary auditory cortex	41	(−48, −16, −1)		10.5
L	superior temporal gyrus, primrary auditory cortex, secondary auditory cortex	41, 42, 22	(−57, −25, 8)		9.04
R	angular gyrus	39	(57, −58, 35)	25	8.58
Male > Female Videos					
R	postcentral sulcus, superior parietal lobule	2, 5, 7	(27, −46, 53)	211	8.83
R	postcentral sulcus, superior parietal lobule	2, 7	(33, −37, 47)		8.09
R	supramarginal gyrus, postcental sulcus	40, 2	(42, −31, 41)		7.83
R	superior occipital lobe	19	(33, −82, 20)	18	7.18
Homosexual Men					
Female > Male Videos					
L	superior temporal gyrus, primary auditory cortex, posterior insula	22, 41, 42	(−57, −31, 5)	365	12.52
L	superior temporal gyrus, primary auditory cortex	41	(−48, −28, 5)		11.96
L	superior temporal gyrus	22	(−57, −10, −1)		10.65
R	superior temporal gyrus, middle temporal gyrus	22, 42, 41	(63, −19, −4)	318	11.71
R	superior temporal gyrus	22	(63, −25,8)		11.7
R	primary auditory cortex, secondary auditory cortex	42, 41	(48, −19, 5)		9.82
R	supramarginal gyrus	40	(63, −43, 38)	44	8.61
R	angular gyrus	40	(60, −52, 29)		6.82
Male > Female Videos					
R	postcentral gyrus, postcentral sulcus, supramarginal gyrus	3, 1, 2, 40	(30, −34, 47)	663	10.04
R	superior parietal lobule, postcentral gyrus	7, 5, 3, 1, 2	(39, −37, 56)		9.92
R	superior parietal lobule, precuneus, superior occipital lobe, precuneus, cuneus, middle occipital lobe, primary visual cortex	7, 19, 18, 17	(24, −61, 62)		9.33
R	middle frontal gyrus	6	(27, −4, 56)	93	9.82
R	middle frontal gyrus	6	(36, −4, 62)		9.8
R	middle frontal gyrus	6	(36, −4, 53)		9.77
L	primary visual cortex	17	(−12, −82, 8)	30	8.59
R	inferior temporal gyrus, occipitotemporal junction	37	(45, −70, −4)	63	8.2
R	inferior temporal gyrus, lateral occipital gyrus	19	(48, −58, −10)		6.9
L	superior parietal lobule	7	(−21, −58, 56)	104	8.12
L	superior parietal lobule	7	(−30, −49, 56)		7.77
L	postcentral gyrus, postcentral sulcus	3, 1, 2	(−24, −34, 44)		7.7
R	fusiform gyrus	37	(33, −52, −16)	21	7.88
L	middle occipital gyrus	19	(−24, −97, 14)	80	7.17
L	middle occipital gyrus	19	(−39, −79, 11)		7.16
L	middle occipital gyrus, extending into occipitotemporal junction	19, 37	(−36, −79, −1)		6.77
L	superior occipital	19	(−24, −82, 32)	10	6.96

Results are presented separately for each orientation group. L = left, R = right, BA = Brodmann area. Coordinates are in MNI space. All clusters were significant with p < 0.05 FWE corrections.
